# The Dissemination of Tubercle Bacilli by Cows in Coughing a Possible Source of Contagion1Read before the Pathological Society of Philadelphia, November 8, 1900.

**Published:** 1901-01

**Authors:** Mazyck P. Ravenel

**Affiliations:** The Laboratory of the State Livestock Sanitary Board of Pennsylvania


### THE DISSEMINATION OF TUBERCLE BACILLI BY COWS IN COUGHING A POSSIBLE SOURCE OF CONTAGION.[1]

By Mazyck P. Ravenel, M.D.

(*From the Laboratory of the State Livestock Sanitary Board of Pennsylvania.*)

At the present day it is the general opinion of investigators and physicians that the chief method of the dissemination of tuberculosis is the inhalation of dried tuberculous sputum, which becomes pulverized and is carried about by currents of air or raised from its resting place in the cracks of floors, for instance, through sweeping and such like processes. The experiments of Cornet, made under the direction of Koch, are strongly corroborative of this opinion. The habit of indiscriminate spitting by persons suffering from consumption has until very recently been accepted, then, as the chief means by which the contagion was spread, and such, indeed, is the general opinion held to-day. Flügge has recently taken exception to this opinion, and holds that the spread of tuberculosis is due mainly to the inhalation of minute particles of sputum which is brought to a state of fine division during the act of coughing. He believes that these minute particles float in the air for some time, and may be carried by very slight currents. He brought forward as confirmatory of this opinion experiments by himself and his assistant, which are now well-known. In this country Klebs has by similar experiments shown that during the act of coughing minute particles of sputum, oftentimes containing tubercle bacilli, are thrown out. Dr. Curry, of Boston,[2] at the

[1] Read before the Pathological Society of Philadelphia, November 8, 1900.
[2] Boston Medical and Surgical Journal, October, 1898, vol. cxxxix., No 15.

suggestion of Klebs, carried out experiments in twelve cases of tuberculosis with a view of determining what degree of danger there might be from this source. He examined the mouth fluid of patients whose sputum revealed the presence of tubercle bacilli, and in nine out of twelve cases the bacilli were found at some time during the day, though usually in very small numbers. In three of the cases many bacilli were found at nearly every examination. As a rule, they were most plentiful in the early morning. He also suspended glass plates before these patients at distances of from one to three feet. By this method one-half of the cases gave negative results, all of which, however, had no cough and were in the habit of keeping the lips closed during coughing. Of the six patients which gave positive results, all had a loud cough and kept the mouth open during coughing. He concludes from his experiments that there is not only a possible but even probable danger from this source, but he considers that Flügge has greatly exaggerated this danger. He very aptly calls attention to a point which seems to have escaped Flügge entirely, namely, that these small particles of sputum are likely to rapidly become dry, in which condition, of course, they act just as so much dried infectious sputum. Wherever particles of sputum which contain virulent tubercle bacilli are thrown out a possible source of danger must inevitably be recognized.

In a series of studies having for their object the relation of bovine tuberculosis to human health, I was led to see if it were not possible that cows in the act of coughing would likewise expel small particles of tubercular material rich in tubercle bacilli. The opinion is widely accepted that cows swallow all of their sputum and do not project it to any extent. Various methods for the collection of sputum from the trachea and larynx of cows have been tried, with the idea of using it for diagnostic purposes. Pols proposed the insertion of a canula into the trachea for the collection of mucus, but this has not proved satisfactory. Nocard has suggested the injection of veratrine or eserine in order to increase secretion from the bronchial tube, but this likewise has been unsuccessful. The use of a swab inserted down the trachea by means of a long handle, as proposed by Greffier, has given better results. The method used in my experiments has been much more simple and easily carried out. It consists in the use of an ordinary nose-bag, near the bottom of which is placed a shelf of soft pine wood, which is sterilized by steam heat each time before using. Such a nose-bag may be left on the animal for several hours at a time, the amount of material collected varying greatly in different animals, and in the same animal from day to day. The smallest particles ejected by the cow during the act of coughing adhere to this piece of soft pine wood, which absorbs most of the fluid portion, leaving the more solid particles standing in relief, so that they can be easily detected by the naked eye or by a low magnifying glass. From this they may be removed with a platinum needle to a cover-slip and examined under the microscope. By this means I have been able to detect tubercle bacilli in the bronchial secretions of every tuberculous cow in which it has been tried. In one animal the amount of secretion was exceedingly minute, and even after the nose-bag had been kept on her for three and a half to four hours there would often be only a few particles of matter not larger than the head of a pin, but they were almost always exceedingly rich in tubercle bacilli. In this way what might be expected theoretically has been practically demonstrated, namely, that in the act of coughing cows as well as men atomize, so to speak, their sputum and project it into the air in minute particles, which may float for a considerable period of time. Secretion collected in this way has been inoculated into the peritoneal cavity of guinea-pigs, and even when the bacilli could be demonstrated under the microscope a considerable proportion of positive results have been obtained. I do not mean to advocate this method as a means of diagnosis, although my experiments warrant me in believing that tubercle bacilli can always be found in the sputum of tuberculous animals at some time, but doubtless in early cases it would require a large number of examinations, and with the well-established use of tuberculin it would seem unnecessary to resort to this means. The danger of infection by means of this atomized sputum, as far as mankind goes, is confined practically to those in constant contact with the animals, but for other animals in the same stable these particles must be considered as a source of danger.

Of thirty-four examinations carried out on five different animals tubercle bacilli were detected by microscopical examination twenty times. The number of bacilli found varied greatly, but one of the five animals coughed up a tenacious mucus in which the numbers approached those seen in human sputum from advanced cases.

During a period of time extending over forty-three days mucus from two cows was collected by means of the nose-bag on eighteen days and inoculated into the peritoneal cavity of forty-five guinea-pigs. Of these, twenty-three died within a few days, most of them from peritonitis, at a period too early for the development of tubercular lesions. Subtracting these, we have remaining twenty-two animals, eleven of which, or 50 per cent., became markedly tuberculous.

By means of a special nose-bag guinea-pigs were exposed directly to the breath of cows in whose sputum tubercle bacilli had been found. Fourteen pigs were exposed for varying periods of time, as follows:

| | | | | | |
|---|---|---|---|---|---|
| 2 guinea-pigs | for | 2 | hours | on | 1 day. |
| 2 | " | " | $2\frac{1}{2}$ | " | 1 " |
| 2 | " | " | 3 | " | 1 " |
| 2 | " | " | 3 | " | 1 " |
| 2 | " | " | 5 | " | 2 days. |
| 2 | " | " | 15 | " | 5 " |
| 2 | " | " | 27 | " | 9 " |

These animals were killed after several weeks, but no evidence of tuberculosis could be detected in any of them.

The cows on which the examinations were carried out were all marked cases of tuberculosis, though only one of them was in the last stages of the disease. One animal, which gave a large proportion of positive results, lived for more than two years after the experiment.

